# FISH mapping of Philadelphia negative *BCR/ABL1 *positive CML

**DOI:** 10.1186/1755-8166-1-14

**Published:** 2008-07-18

**Authors:** Anna Virgili, Diana Brazma, Alistair G Reid, Julie Howard-Reeves, Mikel Valgañón, Anastasios Chanalaris, Valeria AS De Melo, David Marin, Jane F Apperley, Colin Grace, Ellie P Nacheva

**Affiliations:** 1 Molecular Cytogenetics, Academic Haematology, Royal Free and UCL Medical School, Rowland Hill Street, London, NW3 2PF, UK; 2 Imperial College, Faculty Medicine, Hammersmith Hospital, Dept of Haematology, Du Cane Road, London, W12 ONN, UK

## Abstract

**Background:**

Chronic myeloid leukaemia (CML) is a haematopoietic stem cell disorder, almost always characterized by the presence of the Philadelphia chromosome (Ph), usually due to t(9;22)(q34;q11) or its variants. The Ph results in the formation of the *BCR/ABL1 *fusion gene, which is a constitutively activated tyrosine kinase. Around 1% of CML patients appear to have a Ph negative karyotype but carry a cryptic *BCR/ABL1 *fusion that can be located by fluorescence *in situ *hybridisation (FISH) at chromosome 22q11, 9q34 or a third chromosome. Here we present FISH mapping data of *BCR *and *ABL1 *flanking regions and associated chromosomal rearrangements in 9 Ph negative *BCR/ABL1 *positive CML patients plus the cell line CML-T1.

**Results:**

*BCR/ABL1 *was located at 9q34 in 3 patients, 22q11 in 5 patients and CML-T1 and 22p11 in 1 patient. In 3 of 6 cases with the fusion at 22q11 a distal breakpoint cluster was found within a 280 Kb region containing the *RAPGEF1 *gene, while in another patient and the CML-T1 the distal breakpoint fell within a single BAC clone containing the 3' *RXRA *gene. Two cases had a duplication of the masked Ph while genomic deletions of the flanking regions were identified in 3 cases. Even more complex rearrangements were found in 3 further cases.

**Conclusion:**

*BCR/ABL1 *formation resulted from a direct insertion (one step mechanism) in 6 patients and CML-T1, while in 3 patients the fusion gene originated from a sequence of rearrangements (multiple steps). The presence of different rearrangements of both 9q34 and 22q11 regions highlights the genetic heterogeneity of this subgroup of CML. Future studies should be performed to confirm the presence of true breakpoint hot spots and assess their implications in Ph negative *BCR/ABL1 *positive CML.

## Background

Chronic myeloid leukaemia (CML) is a pluripotent haematopoietic stem cell disorder defined by expression of the *BCR/ABL1 *fusion gene, a constitutively activated tyrosine kinase, harbored by the Philadelphia chromosome (Ph), which is a result of a t(9;22)(q34;q11) or a related variant translocation [1]. In ~1% of the CML patients the bone marrow cells appear to be Ph negative by G-banding, although the *BCR/ABL1 *fusion gene can be identified by molecular means and located by fluorescence *in situ *hybridisation (FISH) on chromosome 22q11, 9q34 or even a third chromosome. The biology and clinical significance of the genetic rearrangements in Ph negative *BCR/ABL1 *positive disease have been widely discussed following the first descriptions [2-5]. Two mechanisms for the formation of the chimeric gene in masked Ph positive cells have been postulated: either by insertion of *ABL1 *into the *BCR *region (or vice versa) or by a multiple step model where a classical t(9;22) is followed by a translocation of both products and/or another autosome, thereby restoring the normal chromosome morphology. In both instances, more than the 2 breaks associated with classical t(9;22) are implicated. Although as early as 1990 Morris et al. [6] provided evidence that the insertion involves additional sequences distal to the *3' ABL1 *site, the extent of the genomic rearrangements in this form of CML are unknown.

In view of the few studies published with a precise map of the insertions [7-10], we aimed to construct an accurate map of the insertions in the cell line CML-T1 [11] and 9 patients with Ph negative *BCR/ABL1 *positive CML using a range of FISH probes obtained from BAC clones. The fusion gene was identified at 9q34 (3 patients), 22q11 (5 patients and CML-T1) and 22p11 (3 patients), resulting in relocation of sequences well in excess of either 3' *ABL *or 5' *BCR *by means of a direct insertion (6 patients and CML-T1) or a sequence of events (3 patients). Recurrent distal breakpoints were found at the regions of *RAPGEF1 *and *RXRA *genes.

## Methods

Nine archival bone marrow chromosome preparation samples of CML patients (7 females and 2 males) with Ph negative *BCR/ABL1 *positive disease collected from Hammersmith and Royal Free Hospitals (London, UK), together with the cell line CML-T1, were investigated (Table 1). All samples tested positive for *BCR/ABL1 *fusion by PCR. Investigations were carried out on bone marrow samples obtained at presentation. G-banding and molecular cytogenetic analysis, including chromosome painting and FISH mapping with locus specific probes, were performed following protocols in routine use [12]. A minimum of 25 metaphase and over 100 interphase cells after short term in vitro culturing were analysed and results described following ISCN (2005). Five of the samples in this cohort (cases 1–3, 7 & 8) were part of another study [13].

**Table 1:** Characteristics of the samples.

**Case no.**	**Sample type**	**Karyotype**	***BCR/ABL1 *fusion**
1	Bone marrow	46, XX [20]	e13a2
2	Bone marrow	46, XX [20]	e14a2
3	Bone marrow	46, XX [20]	e13a2
4	Bone marrow	46, XX [20]	NA
5	Cell line (CML-T1)	92 < 4n > XXX, t(6;7)(q24;q35)x2, del(11) (q?22.3)x2[12]/46, XX, idem[18]	e13a2
6	Bone marrow	46, XX [20]	e13a2
7	Bone marrow	46, XX [20]	e13a2/e14a2
8	Bone marrow	46, XX [20]	e13a2
9	Bone marrow	46, XX [20]	NA
10	Bone marrow	46, XX [20]	NA

NA: Not available

In all samples, FISH with the commercially available LSI *BCR/ABL1 *Dual Color, Dual Fusion Translocation Probe ("D-FISH", Vysis, Downers Grove, IL, USA) was initially performed using manufacturer's protocol to identify the chromosome location of the *BCR/ABL1 *fusion gene. FISH mapping was carried out with Bacterial Artificial Chromosomes (BAC) clones obtained from the BACPAC Resources Center (Children's Hospital Oakland Research Institute, Oakland, CA, USA), the Sanger Centre (Cambridge, UK) and Invitrogen (Paisley, UK) (Table 2). Clones were grown in Luria-Bertani medium with Chloramphenicol (20 μg/ml), BAC DNA extracted with a QIAGEN Large-Construct Kit (Qiagen, West Sussex, UK) and directly labelled with either Spectrum Orange or Spectrum Green dUTPs with a Nick Translation Kit (Vysis). BAC DNA from chromosomes 9 and 22 belonging to the Human 32 K Clone Set from the BACPAC Resources Center was amplified with a GenomePlex Single Cell Whole Genome Amplification Kit (Sigma-Aldrich, Dorset, UK) and labelled as described before.

**Table 2:** Summary of the BAC clones used for FISH analysis, their genomic address and source.

**BAC Clone**	**Chromosome** ** address**	**Start**	**End**	**Source**
RP11-17O4	9q34.1	129,342,001	129,449,609	Sanger
RP11-138E2	9q34.1	129,759,745	129,938,526	Sanger
RP11-88G17	9q34.1	129,970,404	130,152,614	Sanger
RP11-57C19	9q34.1	130,510,099	130,683,561	Invitrogen
RP11-83J21	9q34.1	130,681,562	130,858,027	BACPAC
RP11-544A12	9q34.1	130,994,805	131,191,826	Invitrogen
RP11-643E14	9q34.1	131,186,189	131,363,951	Sanger
CTD-2107G12	9q34.1	131,334,305	131,468,329	BACPAC (32K Clone Set)
RP11-40A7	9q34.1	131,422,266	131,599,379	BACPAC
RP11-323H21	9q34.1	131,659,962	131,844,139	BACPAC
CTD-2505O5	9q34.1	131,842,073	131,989,317	BACPAC (32K Clone Set)
RP11-666F23	9q34.1	132,014,407	132,158,747	BACPAC (32K Clone Set)
RP11-81P5	9q34.1	132,115,450	132,308,214	BACPAC
RP11-326L24	9q34.1-q34.2	132,884,992	133,078,984	Sanger
RP11-153P4	9q34.2	133,571,299	133,750,426	Sanger
RP11-145E17	9q34.2	134,270,752	134,427,189	Sanger
RP11-92B21	9q34.2	134,517,511	134,693,859	Sanger
RP11-751H16	9q34.3	134,857,931	135,039,874	BACPAC (32K Clone Set)
RP11-413M3	9q34.3	136,526,666	136,715,127	Sanger
RP11-424E7	9q34.3	138,161,638	138,393,244	BACPAC (32 K Clone Set)
RP11-61N10	22q11.2	21,728,291	21,917,898	BACPAC
RP11-164N13	22q11.2	21,892,457	22,086,126	BACPAC
RP11-529P21	22q11.2	22,073,257	22,227,046	BACPAC (32 K Clone Set)
RP11-223O10	22q11.2	22,203,038	22,369,574	BACPAC (32 K Clone Set)
RP11-698L6	22q11.2	22,333,501	22,518,106	BACPAC (32 K Clone Set)
RP11-594L10	22q11.2	22,725,284	22,866,905	BACPAC (32 K Clone Set)
RP11-446O3	22q11.2	22,846,809	23,014,392	BACPAC (32 K Clone Set)
RP11-765G14	22q11.2	23,590,296	23,758,574	Invitrogen

BAC RP11-164N13 was used to target *BCR *gene. Since this BAC covers both major and minor *BCR *breakpoints, it is found split when *BCR *gene is rearranged. RP11-61N10 covers *BCR *non-coding sequences centromeric to the breakpoint and it was used to identify 5' *BCR *region. RP11-83J21, which covers the 3' end of *ABL1 *incorporating the whole of the coding region, was used to identify the sequences telomeric of the *ABL1 *breakpoint, whereas RP11-57C19 was used to identify the *ABL1 *sequences centromeric of the breakpoint.

The BAC clones and genes were located according to the UCSC database, hg17 (University of California Santa Cruz, CA, USA) [14]. Mapping data for the 32 K human clone set was obtained from the BACPAC Resources Center website [15] and used to assess the size of the sequences found to be rearranged. In addition, sub-telomeric probes directly labelled from the regions of 9q, 22q and 16q were used (Stretton Scientific Ltd, Stretton, UK). All tests were carried out as dual colour, dual probe FISH. Digital imaging and karyotyping were carried out using a SmartCapture and SmartType FISH workstation (Digital Scientific Ltd, Cambridge, UK).

Array CGH analysis (aCGH) was carried out on the cell line CML-T1. The aCGH was performed using two platforms – 1 Mb BAC clone chip (SGI2600) [16] and oligo-nucleotide (105 K Agilent) [17] following manifacturer's protocol, while data processing and presentations were carried out using 'in house' software as reported [18,19].

## Results

**Figure 1:**
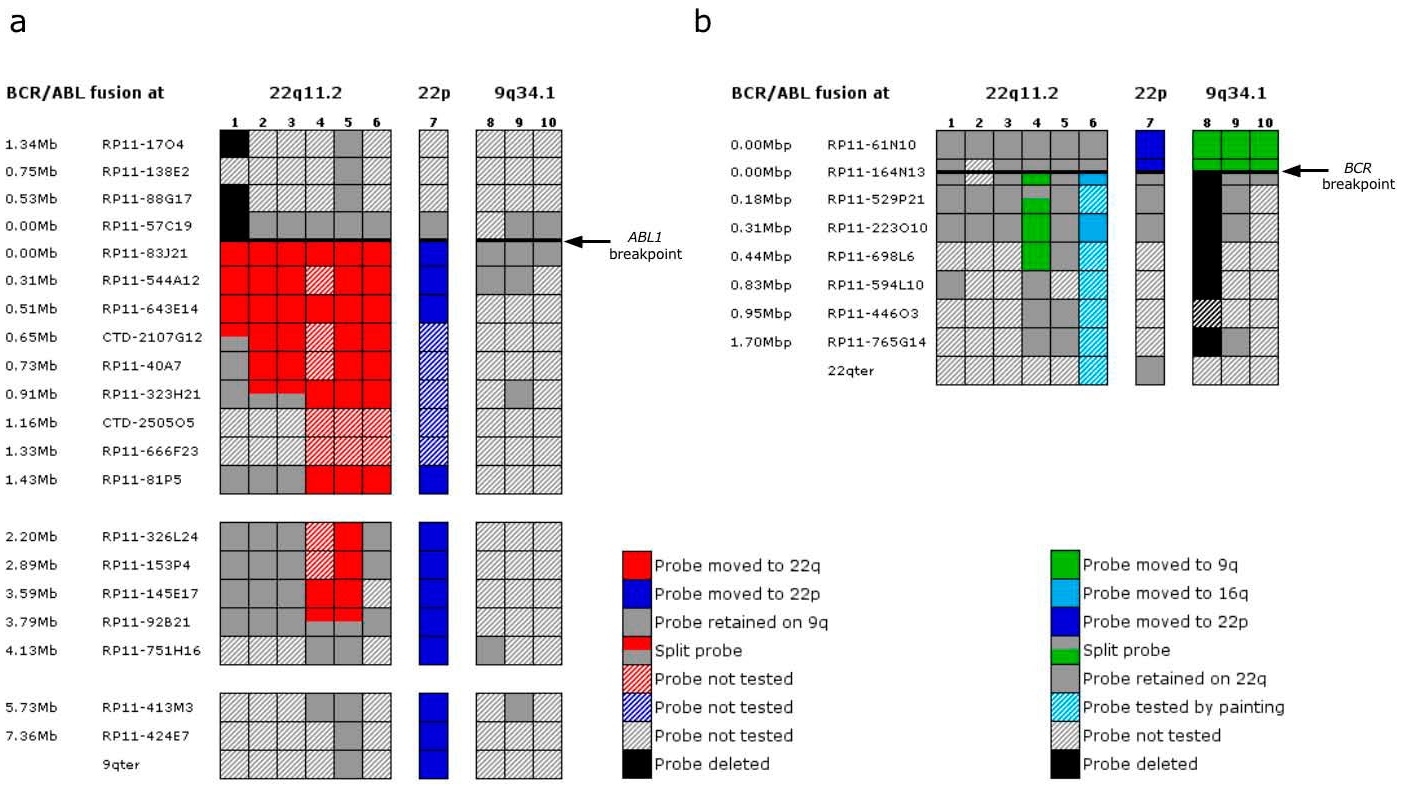
**Summary of the FISH mapping carried out on Ph negative *BCR/ABL1 *positive bone marrow cells from 9 CML patients and the cell line CML-T1**. Header row shows the location of *BCR/ABL1 *fusion: 22q11.2 (cases no 1–6), 22p (case 7) and 9q34.1 (cases 8–10). (a) Map of the 9q34.1-qter region with coloured squares (key on the right) indicating the location or deletion of the BAC clones used for FISH analysis (approximate genomic distances to the breakpoint and names of the BAC clones on the left). A thick black horizontal line presents the *ABL1 *breakpoint, which is encompassed by the clones RP11-57C19 and RP11-83J21. When a breakpoint falls within a BAC, the probe gives a split signal in two different locations. (b) Map of the 22q11.2 region with coloured squares (key below) indicating the location or deletion of the BAC clones tested (genomic distances and names of the clones on the left). A thick black horizontal line presents the *BCR *breakpoint, which falls within the BAC clone RP11-164N13.

A summary of the molecular cytogenetic investigations carried out on *BCR/ABL1 *positive samples from 9 CML patients with normal bone marrow (BM) karyotype as well as the cell line CML-T1 with masked Ph chromosome is presented Figure 1.

### FISH analysis using commercial *BCR/ABL1 *D-FISH probe (Vysis)

The *BCR/ABL1 *D-FISH probe (Vysis) contains sequences covering both genes labelled in different colours, so that rearrangements affecting them can be visualised at chromosome level. The *ABL1 *probe (red) targets a 650 Kb region of 9q34.1 including the whole of the *ABL1 *gene (173.8 Kb), thus spanning the common breakpoint, and extends 5' of *ABL1 *to incorporate the *ASS *gene. The *BCR *probe (green) is represented by two 600 Kb regions of 22q11.2 separated by a 300 Kb gap, one of the regions covering the entire *BCR *gene and extending 5' of it in order to include the *IGLV *gene, and the other starting 300 Kb telomeric of *BCR *and ending at 900 Kb 3' of the gene. The application of this probe revealed the *BCR/ABL1 *fusion at three different chromosome sites: at 22q11.2 (samples no 1–6), at 22p11 (sample no 7) and at 9q34 (samples no 8–10). There was evidence for the formation of the reciprocal *ABL1/BCR *fusion in just one patient (no 4), which showed 2 fusion signals at der(22) and der(9). In the remaining cases only 1 fusion signal was observed, irrespective of the fusion gene location.

In addition, D-FISH also revealed the presence of different clones with further rearrangements in CML-T1 and patients no 1 and 3. CML-T1 was found to harbour 3 clones: i) 7 out of 20 metaphases (35%) showed a 2 red 2 fusion signal pattern typical of diploid cells with duplication of the masked Ph and no normal 22 homologue (loss of the green signal); ii) 7 out of 20 metaphases (35%) showed a 4 red 4 fusion pattern for tetraploid cells with duplication of the masked Ph and no normal 22; iii) 6 out of 20 metaphases (30%) showed a 2 red 4 fusion pattern for tetraploid cells with duplication of the masked Ph, no normal 22 and deletion of 5' *ABL1 *(loss of two red signal). Patient no 3 had also developed a sub-clone with a duplication of the masked Ph and loss of the normal 22 homologue (2 red 2 fusion D-FISH pattern), as seen in 4 out of 20 metaphases (20%). On the other hand, D-FISH in patient no 1 showed a 1 red 1 green 1 fusion signal pattern, uncovering a deletion of sequences centromeric to *ABL1 *at the der(9)ins(22;9) in all cells screened (loss of 1 red signal).

### Patients with *BCR/ABL1 *fusion residing on chromosome 22q11.2

The BAC clone RP11-83J21 covering 3' *ABL1 *moved to 22q11 in 6 cases (no 1–6), whereas RP11-57C19 remained at der(9) in all but one (no 1, Figure 1). In patient no 1, we identified a cryptic genome loss at der(9) of at least 1.34 Mb in all cells. The sequences deleted, centromeric of *ABL1 *breakpoint, were covered by the BAC clones RP11-57C19 to RP11-17O4. However, it was not possible to assess the full extend of the deletion due to lack of material.

**Figure 2:**
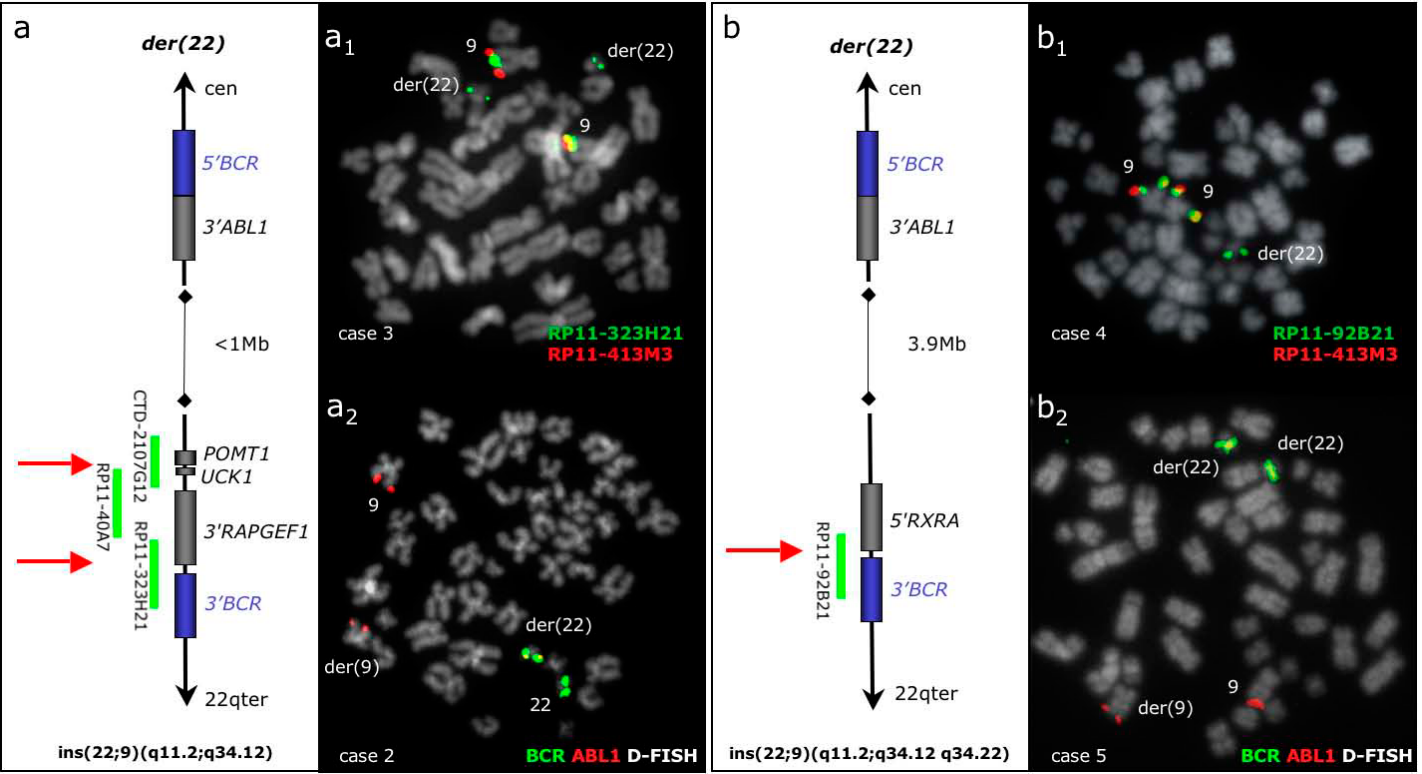
***BCR/ABL1 *fusion at 22q11.2: "small" and "large" insertions with recurrent distal breakpoints**. (a) Diagram showing the small size ins(22;9)(q11;q34.1q34.1) seen in 3 patients (no 1–3). The BAC clones covering the distal breakpoint region are presented with green lines. The *ABL1 *breakpoint marks the proximal boundary of the insertion (< 1 Mb) while the distal breakpoint (shown by red arrows) falls within a 280 Kb breakpoint cluster housing the *UCK1, POMT1 *and *RAPGEF1 *genes. (a_1_) A representative metaphase cell in patient no 3 with co-hybridization of FISH probes RP11-323H21 and RP11-413M3, showing a split signal from RP11-323H21 (green signals at both chromosome 9 homologues and masked Ph) and duplication of the masked Ph (green signals on 2 masked Ph). (a_2_) *BCR/ABL1 *D-FISH (Vysis) in patient no 2, showing the absence of green signal at der(9). (b) Diagram showing the large size ins(22;9)(q11.2;q34.1q34.2) seen in patient no 4 and CML-T1. The *ABL1 *breakpoint marks the proximal boundary of the 3.9 Mb insertion, while the distal breakpoint lies within the clone RP11-92B21 (red arrow). (b_1_) A representative metaphase cell in patient no 4 with co-hybridization of FISH probes RP11-92B21 and RP11-413M3, showing a split signal from RP11-92B21 (green signal at both chromosome 9 homologues and masked Ph). (b_2_) *BCR/ABL1 *D-FISH (Vysis) in CML-T1, showing absence of green signal at der(9) and duplication of the masked Ph (two fusion signals).

In all 6 cases the inserted 9q34 sequences exceeded the 3' boundaries of the *ABL1 *gene and estimated to be 720 Kb long in 1 case (no 1), 1 Mb in 2 cases (no 2–3), between 1.6 Mb and 2.2 Mb in 1 case (no 6) and 3.9 Mb in 2 cases (no 4–5). The estimated sizes of the insertions were calculated based on the information of the BAC clones chromosome location available at the UCSC database (genome build 35). In cases no 1–5 the insertions were found to stretch distally falling into two sub-groups: a "small" insertion (720 Kb–1 Mb) and a "large" insertion (3.9 Mb) (Figure 2). The distal boundary of the "small" insertion seen in 3 patients (no 1–3) fell within a region covered by 3 overlapping BAC clones (CTD-2107G12, RP11-40A7 and RP11-323H21), thus forming a breakpoint cluster (Figure 2) which was estimated to be 280 Kb long and found to house several genes: *POMT1*, *UCK1 *and *RAPGEF1*. In further two samples (patient no 4 and CML-T1) the inserted material was found to be larger (estimated size of 3.9 Mb) and the distal breakpoint fell within a single BAC clone (RP11-92B21) containing the 3' end of the *RXRA *gene (Figure 2).

**Figure 3:**
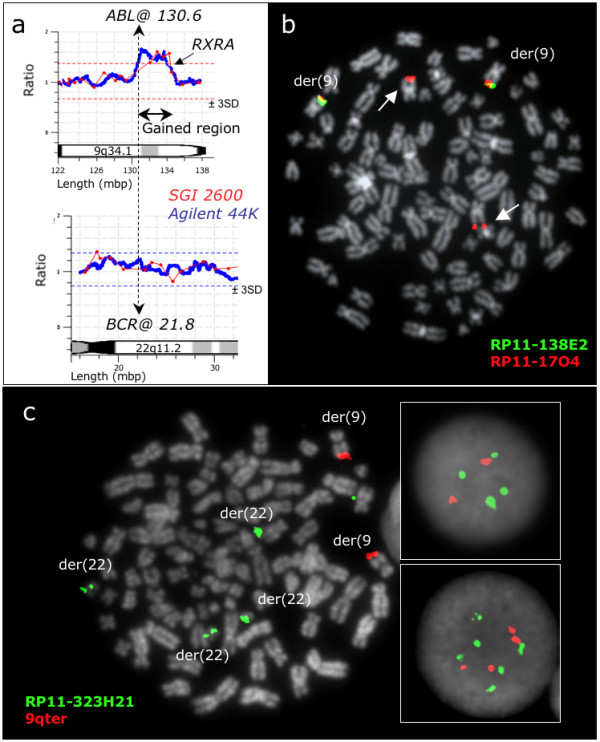
**Gains and deletions in the Ph negative *BCR/ABL1 *positive cell line CML-T1**. (a) Array CGH reveals gains of sequences downstream of the *ABL1 *breakpoint. The genome profile of the 9q34.1-qter region is shown at the top and the 22q11.2-2 region is presented at the bottom, aligned at the *ABL1 *and *BCR *breakpoints (vertical dashed line). Results of the SGI2600 BAC chip are shown in red and 44 K Agilent oligonucleotide in blue. Both BAC and oligo array detect a gain of the 9q34 sequences proximally flanked by the *ABL1 *breakpoint and distally by the *RXRA *gene. (b) A representative tetraploid metaphase cell with co-hybridization of FISH probes RP11-138E2 and RP11-17O4, showing the proximal breakpoint of the 9q34 deletion arisen in this sub-clone (the arrows show the two missing green signals from RP11-138E2). (c) A representative tetraploid metaphase cell with co-hybridization of RP11-323H21 and a 9q sub-telomeric probe (Stretton), showing the duplication of the masked Ph and that the genomic loss affects not the der(9) but the "normal" homologue (the 2 green and 2 red signals from the two normal 9 are missing). The top box on the right shows 4 green, 2 red signals as seen in interphase tetraploid cells with deletion, while the bottom box on the right shows 6 green, 4 red signals as seen in the interphase tetraploid cells without deletion.

High-resolution aCGH analysis of the cell line CML-T1 identified a gain at 9q34.1 starting at *ABL1 *breakpoint at 130.6 and covering 3.9Mb in 3' direction until the distal part of the *RXRA *gene at 134.5, confirming the FISH mapping data (Figure 3). This gain resulted from the duplication of the der(22)ins(22;9) seen in all CML-T1 cells, which was always accompanied by loss of the normal 22 homologue. Furthermore, use of D-FISH with CML-T1 had previously revealed a deletion of 5' *ABL1 *in a tetraploid sub-clone. We mapped the length of the deletion, which was found to be 8.7 Mb long (from RP11-138E2 to 9q telomere) and affect not the der(9)ins(22;9) but the "normal" 9. This was demonstrated by FISH when co-hybridizing a BAC clone found within the insert (in green) and a BAC from chromosome 9 centromeric or telomeric to the inserted sequences (in red). Tetraploid cells with a deletion displayed 2 red signals from two chromosome 9 and 4 green signals from the 4 masked Ph chromosomes. Therefore not only two red signals were deleted, but also the 2 green signals from the "normal" 9 were missing, demonstrating that the loss had occurred at the "normal" homologues.

**Figure 4:**
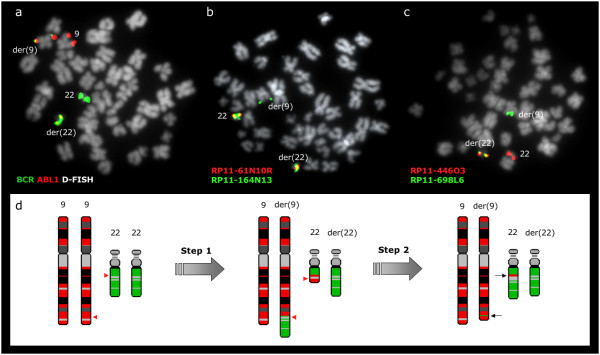
***BCR/ABL1 *at 22q11.2 in patient no 4 results from a multiple step mechanism**. (a) *BCR/ABL1 *D-FISH probe (Vysis) showing 1 red, 1 green, and 2 fusion signals. The presence of both BCR/ABL1 and ABL1/BCR fusion genes is an evidence of an initial t(9;22)(q34;q11.2). (b) A representative metaphase cell with co-hybridization of FISH probes RP11-61N10 and RP11-164N13, showing a split signal from RP11-164N13. Thus, the proximal boundary of the 22q11.2 sequences identified within the structure of the der(9) chromosome coincides with the *BCR *breakpoint. (c) A representative metaphase cell with co-hybridization of FISH probes RP11-698L6 and RP11-446O3. RP11-698L6 is identified at der(9) while RP11-446O3 is found at der(22). The distal breakpoint of the 22q11.2 fragment lies between these two BAC clones. (d) Schematic representation of the multiple step rearrangement with chromosomes 9 in red and 22 in green. Red arrowheads show the breakpoints. The presence of both 9q34 sequences inserted on der(22) and 22q11.2 sequences inserted on der(9) (black arrows) can be explained by two consecutive translocations: an initial t(9;22)(q34;q11.2) followed by a second reciprocal translocation between the two products, with breakpoints distal to both BCR/ABL1 and ABL1/BCR fusion genes.

Regarding *BCR *flanking regions, all probes tested from chromosome 22 remained at their original locations in cases no 1, 2, 3 and 5. However, in case no 4, sequences distal to the *BCR *breakpoint and housed by the BAC clones RP11-164N13, RP11-529P21, RP11-223O10 and RP11-698L6 were found embedded within the der(9) chromosome at the 9q34 region (Figure 4). Both RP11-164N13 and RP11-529P21 gave a split signal pattern being found at the masked Ph and derivative 9, apart from the normal 22. The distal breakpoint of the 22 sequences found at der(9) fell between RP11-698L6 and RP11-594L10.

**Figure 5:**
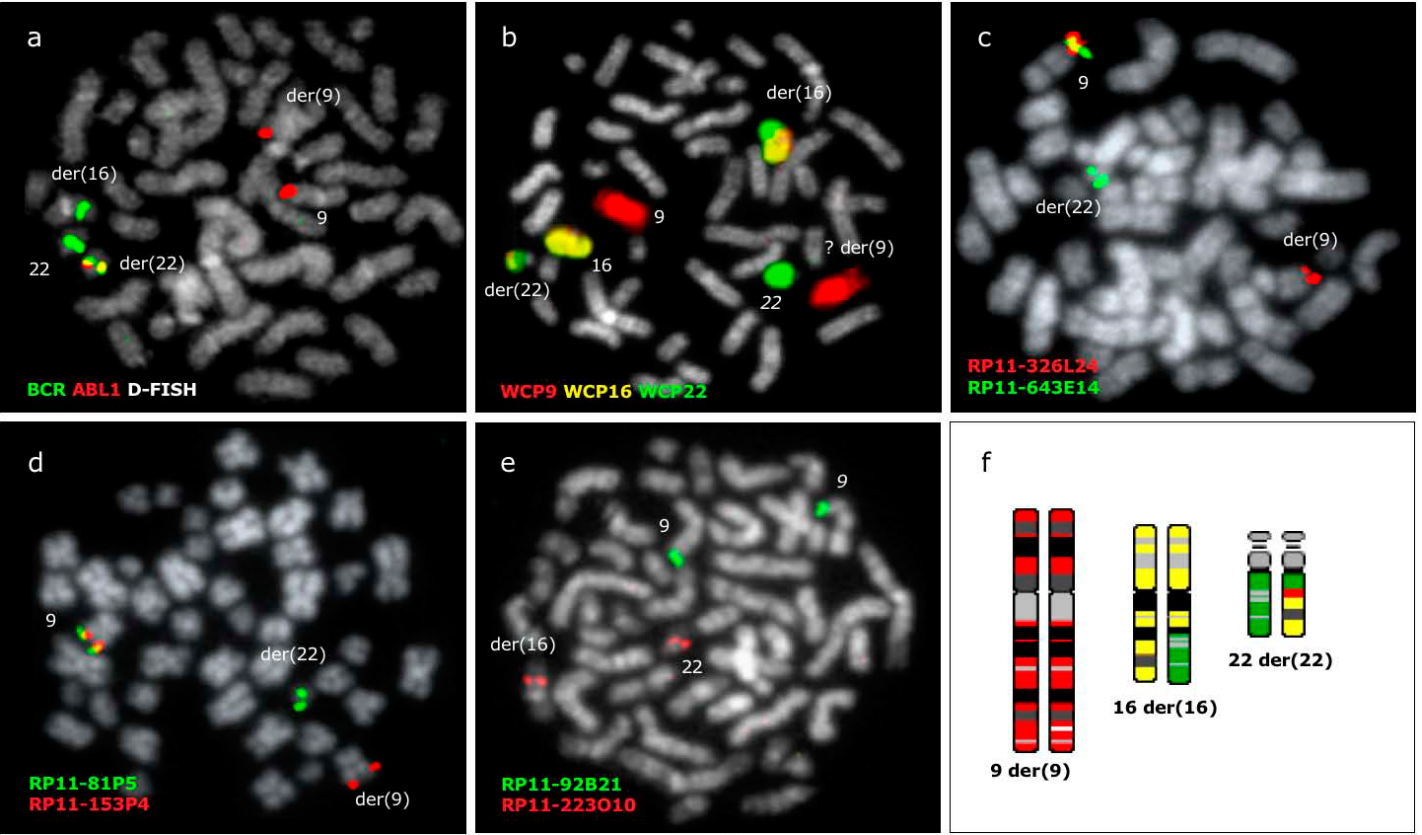
***BCR/ABL1 *resides at 22q11.2 as a result of a cryptic three-way rearrangement between chromosomes 9, 22 and 16 in patient no 6**. (a) *BCR/ABL1 *D-FISH probe (Vysis) showing 1 fusion signal at der(22), 2 red signals at 9 and der(9), 1 green signal at 22 and 1 unexpected green signal at der(16). (b) Chromosome painting confirming the presence of a t(9;22;16)(q34;q11;q?13) in an apparently normal G banding karyotype. (c) FISH with co-hybridization of RP11-326L24 and RP11-643E14 locating RP11-643E14 at the masked Ph while RP11-326L24 is retained at the der(9). (d) A representative metaphase cell with co-hybridization of FISH probes RP11-81P5 and RP11-153P4 identifying RP11-81P5 at the masked Ph. The distal breakpoint of the 9q34 insert is therefore flanked by the BAC clones RP11-81P5 and RP11-326L24. (e) A representative metaphase cell with co-hybridization of FISH probes RP11-92B21 and RP11-223O10 showing RP11-223O10 at normal 22 and der(16), thus confirming the relocation of sequences distal to *BCR *breakpoint at chromosome 16. (f) Schematic representation of the three-way rearrangement with chromosomes 9 in red, 16 in yellow and 22 in green; note that the der(22) contains material from all three parties. The presence of 9q34.1 sequences embedded within the masked Ph suggests that the t(9;22;16) could be a result of a two stage event: firstly ins(22;9) followed by translocation between der(22)ins(9;22) and 16q.

Even more complex rearrangements involving a third chromosome were revealed in another patient (no 6) (Figure 5). FISH painting and FISH mapping identified a three way cryptic translocation t(9;22;16)(q34;q11;q?13) and found the presence of 9q34.1 sequences sandwiched in the der(22)(22pter-q22.1::9q34.1::16q?13-qter). The distal breakpoint of the 9q34.1 fragment fell between RP11-81P5 and RP11-326L24 and the insertion was therefore estimated to be 1.6 Mb–2.2 Mb long. All sequences from chromosome 22 distal to the BCR breakpoint were found at chromosome 16, while sequences proximal to the breakpoint remained at 22q11.

### Patient with the *BCR/ABL1 *fusion residing at 22p11

**Figure 6:**
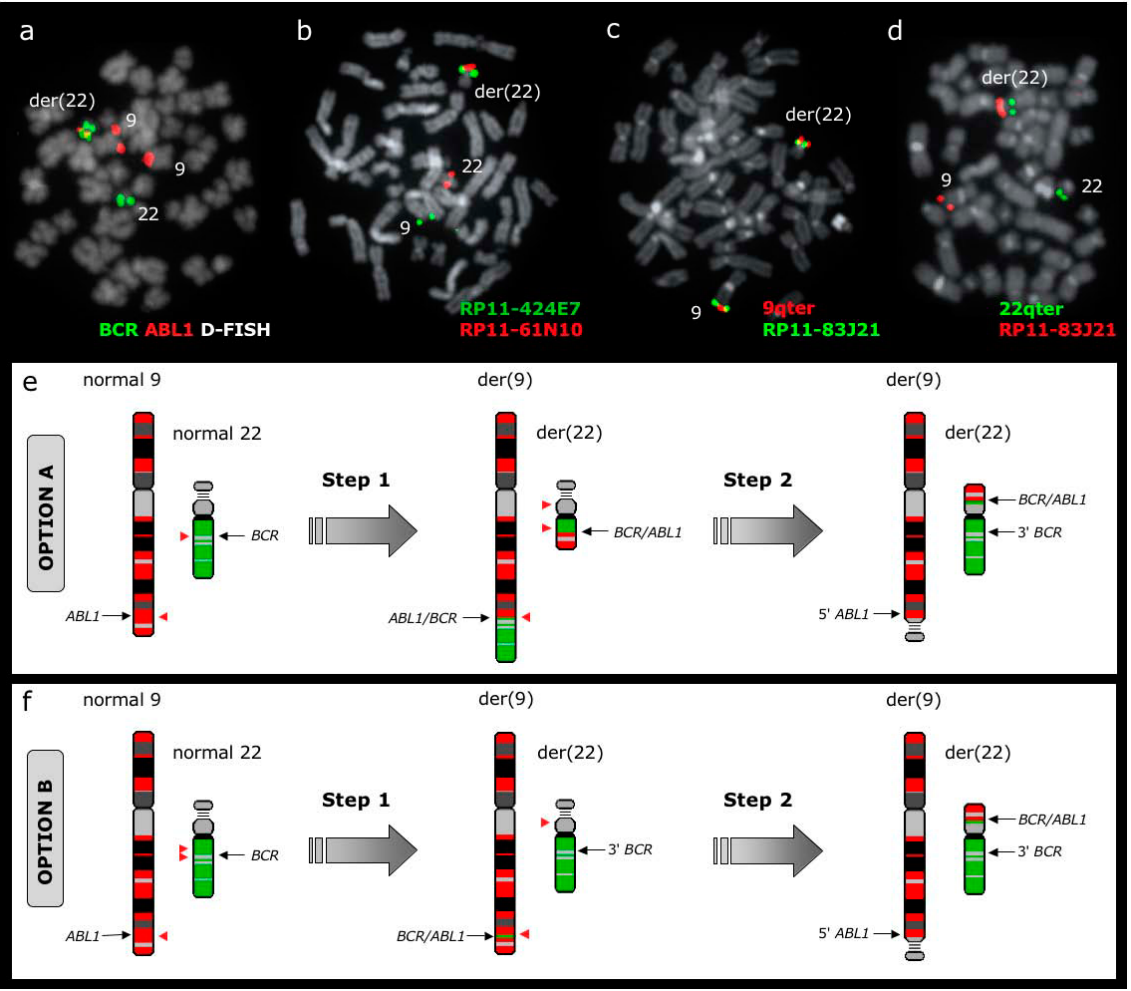
***BCR/ABL1 *fusion resides at 22p11 in patient no 7**. (a) *BCR/ABL1 *D-FISH probe (Vysis) showing a split green signal from *BCR *within the masked Ph. There is only 1 fusion signal located at der(22), another green signal at the same (der22), one green signal at normal 22 and 2 red signals at both chromosomes 9. (b) A representative metaphase cell with co-hybridization of FISH probes RP11-424E7 and RP11-61N10 identifying both probes at the p arm of the masked Ph. (c) A representative metaphase cell with co-hybridization of a 9q sub-telomeric probe (Stretton) and RP11-83J21, showing the presence of the two probes at 22p11 and indicating that all sequences distal to *ABL1 *breakpoint had moved to 22p11. (d) A representative metaphase cell with co-hybridization of a 22q sub-telomeric probe (Stretton) and RP11-83J21, showing that the 22q telomere is retained at its original location. (e) Schematic representation of the events that may have lead to the formation of *BCR/ABL1 *fusion gene and its unusual location at 22p11, with chromosome 9 in red and 22 in green. Sequences distal to *BCR *breakpoint are found at their original location while 5' *BCR *and 9q34 sequences distal to *ABL1 *breakpoint (including the telomere) are relocated at 22p11. This could be explained by an initial t(9;22)(q34;q11) followed by a three-way translocation between 9q34, 22q11 and 22p11, which would require 5 breaks (red arrowheads). (f) The unusual location of BCR/ABL1 at 22p11 could also be a result of an initial ins(9;22)(q34;q11q11) followed by a translocation between der(9) and the p arm of der(22). This sequence of events would need also the same amount of breaks (red arrowheads) and therefore cannot be rule out.

The *BCR/ABL1 *fusion was unexpectedly found at 22p11 in 1 patient (no 7) (Figure 6). FISH with RP11-164N13 showed one signal at the normal chromosome 22 and a split signal at the 22p11 and 22q11 regions from the other homologue. RP11-61N10 was found at the normal 22 and only at the 22p11 region of der(22), thus confirming the location of *BCR/ABL1 *fusion at the p arm of the derivative 22. RP11-529P21, RP11-223O10 and 22qter remained on 22q11, while all probes tested from RP11-83J21 to 9qter were found at 22p11.

### Patients with the *BCR/ABL1 *fusion residing at 9q34.1

**Figure 7:**
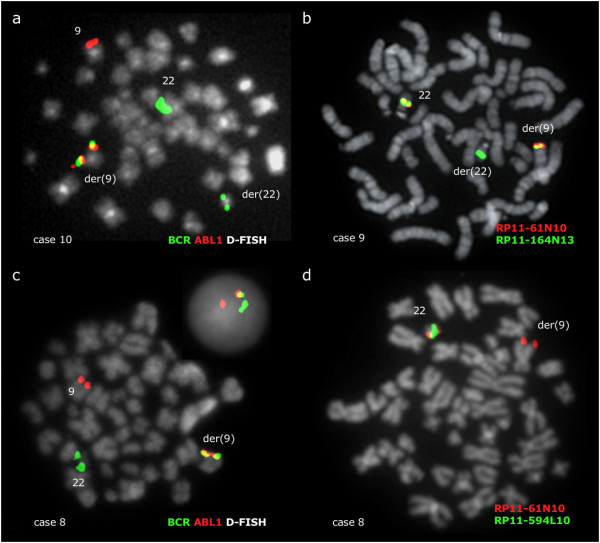
**BCR/ABL1 fusion resides at 9q34.1 in 3 patients**. (a) A representative metaphase cell hybridized with a *BCR/ABL1 *D-FISH probe (Vysis) showing the presence of 1 fusion signal at der(9), 1 red signal at 9 and 2 green signals at both chromosomes 22 as seen in patients no 9 and 10. (b) A representative metaphase cell with co-hybridization of FISH probes RP11-61N10 and RP11-164N13 showing 2 fusion signals at 22 and der(9), plus 1 green signal at der(22), as seen in patients no 9 and 10. RP11-164N13 is therefore split, with the 5' BCR sequences relocated at der(9). (c) *BCR/ABL1 *D-FISH probe (Vysis) in patient no 8 showing 1 fusion signal at der(9), 1 red signal at 9 and 1 green signal at 22. The green signal from der(22) is deleted. (d) A representative metaphase cell with co-hybridization of FISH probes RP11-61N10 and RP11-594L10 giving a fusion signal at normal 22 and one red signal from RP11-61N10 at der(9), confirming that the BCR sequences moved to der(9) are centromeric of the breakpoint. There is again one green signal missing, because the deletion at der(22) includes not only 3' BCR but also at least 1.77 Mb distal to the breakpoint.

The *BCR/ABL1 *fusion was located by FISH at band 9q34.1 in 3 patients (no 8–10) and thought to result from a direct insertion of 22q11.2 sequences. As expected, the telomeric breakpoint was found in all cases within the BAC clone RP11-164N13, which was always seen at both der(9) and der(22) (Figure 7). RP11-61N10 was always found at 9q34, however, due to lack of material it was not possible to assess the proximal breakpoint of the 22q11 insertion. All BACs tested from 9q34 remained at their respective location on chromosome 9 in all 3 patients.

In patient no 8, the insertion was accompanied in all cells by loss of a region at least 1.77 Mb long from 22q11.2 and immediately distal to the *BCR *breakpoint, covered from the 3' end of RP11-164N13 to at least RP11-765G14 (Figure 1).

## Discussion

Since the first description of a Ph negative *BCR/ABL1 *positive CML patient [2], several studies have been published reporting similar cases. Most of them are focused on the presence and location of the *BCR/ABL1 *fusion in CML patients with masked Ph chromosome and commonly achieved by application of commercial FISH probes, which have been proved to be very useful to identify the presence and location of the *BCR/ABL1 *fusion gene in CML patients with no distinguishable Ph chromosome. These studies have established the importance of FISH tests for the diagnosis and therapy monitoring of Ph negative *BCR/ABL1 *positive CML. However, the commercial probes don't provide enough information to understand the mechanisms involved in the formation of the masked Ph chromosome.

We used FISH mapping with BAC probes in order to study the formation of the *BCR/ABL1 *fusion and the underlying genomic rearrangements in 9 CML patients with Ph negative *BCR/ABL1 *positive CML and the cell line CML-T1. The formation of the fusion gene resulted from the relocation of not only the 3' *ABL1 *sequences within the *BCR *region at chromosome 22q11.2 or 5' *BCR *sequences within *ABL1 *region at 9q34.1, but also a considerable amount of flanking material, leaving the chromosome morphology apparently intact.

The fusion gene was located at 9q34.1 in 3 patients, at 22q11.2 in 5 patients and CML-T1, and at 22p11 in another patient. 5 out of the 6 cases with a 9q34 insertion at 22q11 displayed recurrent distal breakpoints that fell within two gene bearer regions. Thus, in 3 patients a common breakpoint cluster of 280 Kb was found. According to UCSC database, this region houses 3 genes: *POMT1 *(protein-O-mannosyltransferase1), *UCK1 *(uridine-cytidine kinase 1) and *RAPGEF1 *(guanine nucleotide releasing factor for *RAP1*; also known as *C3G*). In another patient and CML-T1 the breakpoint fell within a single BAC clone encompassing the 3' end of *RXRA *gene (retinoid × receptor alpha). Although further investigations were not carried out in this study, both *RAPGEF1 *and *RXRA *belong to pathways the disruption of which may be relevant to the evolution of the patients. *RAPGEF1 *has been shown to have transformation suppressor activity towards several oncogenes [20,21] and also interact with *BCR/ABL1 *[22]. *RXRA *belongs to a family of nuclear receptors that target multiple signalling pathways [23] and its downregulation has been showed to be critical for neutrophil granulocytes differentiation [24]. Other studies found *RXRA *to contribute in acute promyelocytic leukaemia transformation [25,26]. Although we have no direct evidence for the immediate involvement of either *RAPGEF1 *or *RXRA *genes, their potential role merits further investigation.

Two other mapping studies that found similar insertions have been published [7,8]. In the first study, the authors used FISH mapping to identify the rearrangements involved in 2 Ph negative CML patients with variant translocations. A 3 Mb insertion from 22q11 into *ABL1 *was identified in 1 patient, while the other had a 9q34 insertion at the *BCR *region with a distal breakpoint falling within the clone RP11-353C22 (genome address: 31,278,002–131,588,248). This result matches with our findings since the latter BAC is located within the same 280 Kb common breakpoint region identified in 3 patients of our study. On the other hand, Valle et al. [7] found a 5.6 Mb insertion of 9q34 sequences into *BCR*. This insertion is larger than the ones identified in our study and was not accompanied by any deletions or other rearrangements.

Deletions of 5' *ABL1 *and/or 3' *BCR *sequences at the der(9) chromosome in patients with classical and variant Ph translocations [27] have been shown to have adverse prognostic value in CML patients treated with interferon [28], although their impact in patients being treated with tyrosine kinase inhibitors is controversial [29-31]. Dual colour, dual fusion translocation FISH probes spanning the *BCR *and *ABL1 *genes are very useful for revealing these events but they have a limited value in interphase nuclei in patients with masked Ph, since often the merging of the 5' *BCR *and 3' *ABL1 *signals by simple insertion leads to an apparent loss of one fusion signal that can be falsely assessed as deletion [13]. Thus, D-FISH (Vysis) in a patient with a direct ins(22;9) gives a 2 red, 1 green, 1 fusion signal pattern, which is the same pattern obtained in case of a typical t(9;22) with deletion of 5' *ABL1 *at der(9). If the patient has a direct ins(9;22) the D-FISH signal pattern is 1 red, 2 green, 1 fusion, which could be mistaken for a typical t(9;22) with deletion of 3' *BCR *at der(9). Furthermore, Ph negative *BCR/ABL1 *positive patients with either a cryptic deletion of 5' *ABL1 *or 3' *BCR *show a 1 red, 1 green, 1 fusion D-FISH pattern, also typical for a t(9;22) with deletion of the reciprocal *ABL1/BCR *fusion. Therefore, when a deletion signal pattern is detected by interphase FISH with a dual colour, dual fusion probe, it is essential to look also at the metaphases in order to be able to differentiate a classical t(9;22) with deletion from a simple insertion.

Batista et al [32] reported a Ph negative *BCR/ABL1 *positive patient with an insertion of *ABL1 *into *BCR *associated with a deletion of the *ASS *– 5' *ABL1 *region. Zagaria et al [8] also reported two cases of CML patients with masked Ph and associated deletions: one patient with a cryptic ins(9;22) and a deletion of 5' *ABL1 *and 3' *BCR *regions; the other patient with a multi-step variant translocation and a deletion of around 400 Kb telomeric of the *ABL1 *gene. In addition to them, De Melo et al [13] identified with commercial triple-colour FISH probes 5' *ABL1 *deletions in 2 patients and 3' *BCR *deletion in 1 patient out of 14 CML patients with masked Ph. Our study confirmed and sized such deletions in 2 patients which where also part of De Melo's cohort. CML-T1 also had a 8.7 Mb deletion of 9q34 material in one of the sub-clones, but in this case the loss was found to affect the homologue not involved in the *BCR/ABL1 *formation.

We identified a duplication of the chromosome 22 harboring the *BCR/ABL1 *fusion accompanied by loss of the normal homologue in 1 out of 9 patients in this study plus the cell line CML-T1. Such duplications of the *BCR/ABL1 *bearing chromosome (either chromosome 22 or 9) seem to be a relatively common event in Ph negative *BCR/ABL1 *positive CML patients, being accompanied by loss of the normal homologue in most of the cases and seen both in chronic phase and blast crisis [33-35].

Regarding the formation of the fusion gene in Ph negative *BCR/ABL1 *positive CML patients, Morris et al [6] suggested two possible mechanisms: a one step model, where *BCR/ABL1 *results from a simple insertion of either 3' *ABL1 *into *BCR *or 5' *BCR *into *ABL *after three genomic breaks; and a multiple step model, with an initial classical t(9;22)(q34;q11) followed by a second translocation of both products and/or a third chromosome, requiring a minimum of 4 genomic breaks.

Our study provided evidence for the existence of both mechanisms implicated in the formation of the fusion gene in Ph negative patients. We found a simple insertion (one step event) in 6 out of 9 patients (no 1–3, 8–10) and CML-T1 (no 5), with no evidence of secondary rearrangements within the regions flanking *BCR *and *ABL1 *breakpoints apart from the accompanying deletions seen in 2 patients and CML-T1. On the other hand, traces of sequential rearrangements indicating a multiple step mechanism were found in 3 patients. Patient no 4 had a 9q34 insertion at chromosome 22 with bits from 22q11 distal to the breakpoint embedded within the der(9), suggesting an initial t(9;22)(q34;q11) followed by further translocation of both products. Patient no 7 carried the *BCR/ABL1 *fusion at 22p11. Sequences downstream of the breakpoint remained at their original location, while 5' *BCR *and all 9q34 sequences distal to *ABL1 *breakpoint (including the telomere) were relocated at 22p11, which could be explained by an initial t(9;22)(q34;q11) followed by a three-way translocation between 9q34, 22q11 and 22p11. This sequence of events would have required 5 breaks (2 for the translocation and 3 for the second one). However, an initial ins(9;22) followed by a reciprocal translocation between 9q34 and 22p11 would also require 5 breaks and therefore cannot be ruled out. Finally, patient no 6 had a three way cryptic t(9;22;16)(q34;q11;q?13) with 9q34 sequences embedded within the der(22), suggesting an initial ins(22;9) followed by a translocation between chromosomes 16 and der(22)ins(22;9). These data show not only that the two mechanisms do happen, but also that they are not excluding options. An example of the latter is patient no 6, where an initial direct ins(22;9) would be part of the spectrum of rearrangements that had restored the normal morphology of the der(22).

## Conclusion

In summary, we found that the *BCR/ABL1 *fusion resulted from relocation of not only the 3' *ABL1 *sequences within *BCR *at 22q11.2 or 5' *BCR *sequences within *ABL *but also a considerable amount of flanking material, with distal recurrent breakpoints of the excised 3' *ABL1 *sequences at *RAPGEF1 *and *RXRA *regions. *BCR/ABL1 *resulted from a direct insertion (one step mechanism) in 6 patients and CML-T1, while in 3 patients the fusion gene was a result of a sequence of events (multiple steps). Finally, the presence of different rearrangements of both 9q34 and 22q11 regions demonstrates the genetic heterogeneity of this subgroup of CML. Future studies should be performed to confirm the presence of true breakpoint hot spots and assess their implications in Ph negative *BCR/ABL1 *positive CML.

## Competing interests

The authors declare that they have no competing interests.

## Authors' contributions

AV carried out the FISH mapping and wrote the manuscript. DB, AC and CG performed the array CGH and qPCR analysis. AR, JH, MV and VDM performed G-banding and initial FISH analysis. DM and JFA provided clinical samples. EN designed the study, supervised its execution and co-participated in the writing of the manuscript. All authors read and approved the final manuscript.

## References

[B1] Melo JV, Barnes DJ (2007). Chronic myeloid leukaemia as a model of disease evolution in human cancer. Nat Rev Cancer.

[B2] Hagemeijer A, de Klein A, Godde-Salz E, Turc-Carel C, Smit EM, van Agthoven AJ, Grosveld GC (1985). Translocation of c-abl to "masked" Ph in chronic myeloid leukemia. Cancer Genet Cytogenet.

[B3] Morris CM, Reeve AE, Fitzgerald PH, Hollings PE, Beard ME, Heaton DC (1986). Genomic diversity correlates with clinical variation in Ph'-negative chronic myeloid leukaemia. Nature.

[B4] Hagemeijer A, Buijs A, Smit E, Janssen B, Creemers GJ, Plas D van der, Grosveld G (1993). Translocation of BCR to chromosome 9: a new cytogenetic variant detected by FISH in two Ph-negative, BCR-positive patients with chronic myeloid leukemia. Genes Chromosomes Cancer.

[B5] Nacheva E, Holloway T, Brown K, Bloxham D, Green AR (1994). Philadelphia-negative chronic myeloid leukaemia: detection by FISH of BCR-ABL fusion gene localized either to chromosome 9 or chromosome 22. Br J Haematol.

[B6] Morris CM, Heisterkamp N, Kennedy MA, Fitzgerald PH, Groffen J (1990). Ph-negative chronic myeloid leukemia: molecular analysis of ABL insertion into M-BCR on chromosome 22. Blood.

[B7] Valle L, Fernandez V, Perez-Pons C, Sanchez FG, Benitez J, Urioste M (2006). Generation of the BCR/ABL fusion gene in a Philadelphia chromosome-negative chronic myeloid leukaemia: insertion of 5.6 Mb of 9q34 into the BCR region of chromosome 22. Hematol Oncol.

[B8] Zagaria A, Anelli L, Albano F, Vicari L, Schiavone EM, Annunziata M, Pane F, Liso V, Rocchi M, Specchia G (2006). Molecular cytogenetic characterization of deletions on der(9) in chronic myelocytic leukemia. Cancer Genet Cytogenet.

[B9] Storlazzi CT, Anelli L, Surace C, Lonoce A, Zagaria A, Nanni M, Curzi P, Rocchi M (2002). Molecular cytogenetic characterization of a complex rearrangement involving chromosomes 9 and 22 in a case of Ph-negative chronic myeloid leukemia. Cancer Genet Cytogenet.

[B10] Todoric-Zivanovic B, Marisavljevic D, Surace C, Cemerikic V, Markovic O, Krtolica K, Tatomirovic Z, Cikota B, Magic Z, Rocchi M (2006). A Ph-negative chronic myeloid leukemia with a complex BCR/ABL rearrangement and a t(6;9)(p21;q34.1). Cancer Genet Cytogenet.

[B11] Kuriyama K, Gale RP, Tomonaga M, Ikeda S, Yao E, Klisak I, Whelan K, Yakir H, Ichimaru M, Sparkes RS, Dreazen O (1989). CML-T1: a cell line derived from T-lymphocyte acute phase of chronic myelogenous leukemia. Blood.

[B12] Gribble SM, Reid AG, Roberts I, Grace C, Green AR, Nacheva EP (2003). Genomic imbalances in CML blast crisis: 8q24.12-q24.13 segment identified as a common region of over-representation. Genes Chromosomes Cancer.

[B13] De Melo VA, Milojkovic D, Marin D, Apperley JF, Nacheva EP, Reid AG (2008). Deletions adjacent to BCR and ABL1 breakpoints occur in a substantial minority of chronic myeloid leukemia patients with masked Philadelphia rearrangements. Cancer Genet Cytogenet.

[B14] UCSC Human Genome Browser Gateway. http://genome.ucsc.edu.

[B15] Human 32K BAC Re-Array. http://bacpac.chori.org/genomicRearrays.php.

[B16] Spectral Genomics. http://www.spectralgenomics.com.

[B17] Agilent. http://www.agilent.com.

[B18] Brazma D, Grace C, Howard J, Melo JV, Holyoke T, Apperley JF, Nacheva EP (2007). Genomic profile of chronic myelogenous leukemia: Imbalances associated with disease progression. Genes Chromosomes Cancer.

[B19] Molecular Cytogenetics of Haematological Disorders. http://www.ucl.ac.uk/leukemia-cytogenetics.

[B20] Guerrero C, Fernandez-Medarde A, Rojas JM, Font de Mora J, Esteban LM, Santos E (1998). Transformation suppressor activity of C3G is independent of its CDC25-homology domain. Oncogene.

[B21] Guerrero C, Martin-Encabo S, Fernandez-Medarde A, Santos E (2004). C3G-mediated suppression of oncogene-induced focus formation in fibroblasts involves inhibition of ERK activation, cyclin A expression and alterations of anchorage-independent growth. Oncogene.

[B22] Cho YJ, Hemmeryckx B, Groffen J, Heisterkamp N (2005). Interaction of Bcr/Abl with C3G, an exchange factor for the small GTPase Rap1, through the adapter protein Crkl. Biochem Biophys Res Commun.

[B23] Ahuja HS, Szanto A, Nagy L, Davies PJ (2003). The retinoid × receptor and its ligands: versatile regulators of metabolic function, cell differentiation and cell death. J Biol Regul Homeost Agents.

[B24] Taschner S, Koesters C, Platzer B, Jorgl A, Ellmeier W, Benesch T, Strobl H (2007). Down-regulation of RXRalpha expression is essential for neutrophil development from granulocyte/monocyte progenitors. Blood.

[B25] Zhu J, Nasr R, Peres L, Riaucoux-Lormiere F, Honore N, Berthier C, Kamashev D, Zhou J, Vitoux D, Lavau C, de The H (2007). RXR is an essential component of the oncogenic PML/RARA complex in vivo. Cancer Cell.

[B26] Zeisig BB, Kwok C, Zelent A, Shankaranarayanan P, Gronemeyer H, Dong S, So CW (2007). Recruitment of RXR by homotetrameric RARalpha fusion proteins is essential for transformation. Cancer Cell.

[B27] Sinclair PB, Nacheva EP, Leversha M, Telford N, Chang J, Reid A, Bench A, Champion K, Huntly B, Green AR (2000). Large deletions at the t(9;22) breakpoint are common and may identify a poor-prognosis subgroup of patients with chronic myeloid leukemia. Blood.

[B28] Huntly BJ, Reid AG, Bench AJ, Campbell LJ, Telford N, Shepherd P, Szer J, Prince HM, Turner P, Grace C, Nacheva EP, Green AR (2001). Deletions of the derivative chromosome 9 occur at the time of the Philadelphia translocation and provide a powerful and independent prognostic indicator in chronic myeloid leukemia. Blood.

[B29] Huntly BJ, Guilhot F, Reid AG, Vassiliou G, Hennig E, Franke C, Byrne J, Brizard A, Niederwieser D, Freeman-Edward J, Cuthbert G, Bown N, Clark RE, Nacheva EP, Green AR, Deininger MW (2003). Imatinib improves but may not fully reverse the poor prognosis of patients with CML with derivative chromosome 9 deletions. Blood.

[B30] Quintas-Cardama A, Kantarjian H, Talpaz M, O'Brien S, Garcia-Manero G, Verstovsek S, Rios MB, Hayes K, Glassman A, Bekele BN, Zhou X, Cortes J (2005). Imatinib mesylate therapy may overcome the poor prognostic significance of deletions of derivative chromosome 9 in patients with chronic myelogenous leukemia. Blood.

[B31] Kreil S, Pfirrmann M, Haferlach C, Waghorn K, Chase A, Hehlmann R, Reiter A, Hochhaus A, Cross NC (2007). Heterogeneous prognostic impact of derivative chromosome 9 deletions in chronic myelogenous leukemia. Blood.

[B32] Batista DA, Hawkins A, Murphy KM, Griffin CA (2005). BCR/ABL rearrangement in two cases of Philadelphia chromosome negative chronic myeloid leukemia: deletion on the derivative chromosome 9 may or not be present. Cancer Genet Cytogenet.

[B33] Costa D, Espinet B, Queralt R, Carrio A, Sole F, Colomer D, Cervantes F, Hernandez JA, Besses C, Campo E (2003). Chimeric BCR/ABL gene detected by fluorescence in situ hybridization in three new cases of Philadelphia chromosome-negative chronic myelocytic leukemia. Cancer Genet Cytogenet.

[B34] Dufva IH, Karle H, Brondum-Nielsen K, Andersen MK, Madsen HO, Johnsen HE (2005). Chronic myeloid leukaemia with BCR-ABL fusion genes located to both chromosomes 9, cyclic leukocytosis and nodal T-lymphoblastic transformation–durable complete remission following imatinib therapy. Leukemia.

[B35] Fugazza G, Garuti A, Marchelli S, Miglino M, Bruzzone R, Gatti AM, Castello S, Sessarego M (2005). Masked Philadelphia chromosome due to atypical BCR/ABL localization on the 9q34 band and duplication of the der(9) in a case of chronic myelogenous leukemia. Cancer Genet Cytogenet.

